# Nonlinear Finite Element Analysis of γ-Graphyne Structures under Shearing

**DOI:** 10.3390/molecules27051729

**Published:** 2022-03-07

**Authors:** Sotirios G. Siampanis, Georgios I. Giannopoulos, Nikos D. Lagaros, Antonios Hatziefremidis, Stelios K. Georgantzinos

**Affiliations:** 1 Department of Aerospace Science and Technology, National and Kapodistrian University of Athens, 34400 Psachna, Greece; ssiampanis@uoa.gr (S.G.S.); ahatzie@uoa.gr (A.H.); 2 General Department, National and Kapodistrian University of Athens, 34400 Psachna, Greece; 3 Department of Mechanical Engineering, School of Engineering, University of Peloponnese, 1 Megalou Alexandrou Street, 26334 Patras, Greece; ggiannopoulos@uop.gr; 4 Institute of Structural Analysis and Antiseismic Research, School of Civil Engineering, National Technical University of Athens, 15780 Zographou, Greece; nlagaros@central.ntua.gr

**Keywords:** γ-graphyne structures, nonlinear finite element analysis, shear, stress–strain

## Abstract

In this study, a nonlinear, spring-based finite element approach is employed in order to predict the nonlinear mechanical response of graphyne structures under shear loading. Based on Morse potential functions, suitable nonlinear spring finite elements are formulated simulating the interatomic interactions of different graphyne types. Specifically, the four well-known types of γ-graphyne, i.e., graphyne-1 also known as graphyne, graphyne-2 also known as graphdiyne, graphyne-3, and graphyne-4 rectangular sheets are numerically investigated applying appropriate boundary conditions representing shear load. The obtained finite element analysis results are employed to calculate the in-plane shear stress–strain behaviour, as well as the corresponding mechanical properties as shear modulus and shear strength. Comparisons of the present graphyne shearing response predictions with other corresponding estimations are performed to validate the present research results.

## 1. Introduction

In recent years, there has been a request to advance the related research area by finding new graphene-like materials and structures at the nanoscale, with similar, if not better, than graphene physical properties and structural characteristics [1,2,3,4,5,6]. The key success behind this development of carbon allotrope technology is based on their outstanding mechanical behaviour characteristics, such as low density, high stiffness, and strength [7,8].

Among the most promising recently explored types of graphene-like nanostructures is the graphyne family of materials [9], which was theoretically studied, perhaps for the very first time, by Narita and Nagai [10], applying first-principles analysis. In a more recent typical theoretical effort using semi-empirical quantum mechanical methods and ab initio calculations, Belenkov et al. [11] found the geometrically optimised structures of graphyne base layers composed of carbon atoms in the hybridised sp and sp^2^ states. In particular, they illustrated the stable molecular structure of seven basic modifications of graphyne—namely, α-, β1-, β2-, β3-, γ1-, γ2-, and γ3-graphyne.

The experimental studies related to graphyne structures available in the literature seem to be limited. In a typical attempt, Ivanovskii [12] thoroughly investigated various types of graphyne and graphdiynes providing basic structural and mechanical properties, determined by experiments or evaluated by ab initio theoretical techniques. Furthermore, Zhang et al. [13] systematically studied the Raman spectra of graphyne and graphdiyne and investigated their performance under mechanical stress and strains. More recently, Chen et al. [14] presented recent advances in graphdiyne concerning its synthesis, functionalisation, and electronic applications.

The main objective of the present study is to predict the nonlinear shear mechanical response of the γ family of graphynes by using a computationally efficient finite element method. Therefore, a detailed literature survey regarding the analysis of graphynes will be presented below in order to make the novel points of the proposed approach distinguishable from other models. A great variety of theoretical–computational calculations may be found in the available literature regarding the mechanical characterisation of graphyne structures. Taking advantage of the generalised computational accuracy of first-principles calculations in approximating the behaviour of complex molecular systems, the mechanical performance and properties of single-atomic-layer graphyne sheets have been already predicted based on the density functional theory (DFT) [15,16,17,18,19]. On another front, molecular dynamics (MD) simulations have evolved into a strong technique that can be applied efficiently to recognise macromolecular structure-to-function relationships. MD has been also utilised [20,21,22,23,24,25,26,27,28] for the investigation of the mechanical response of various graphyne structures. It is worth noting that even though there are several first-principles and MD computations concerning two-dimensional graphynes, only some reports are associated with SM-based approaches for graphynes [29,30,31,32,33,34,35,36]. Still, most of these theoretical analyses, which are grounded on SM, concentrate on the calculation of the linear elastic properties of graphynes. Silvestre et al. [30,31] are perhaps the only researchers who tried to simulate the nonlinear mechanical behaviour of an almost square graphyne-1 sheet of a specific size using SM.

Shear loading is one of the basic loadings of every structure producing shear stress and shear strain. Developing new products, it is particularly useful for the designers the mechanical behaviour of the components under shearing to be predictable. Concerning the response of graphyne structures under shearing, most of the presented studies in the literature are limited to the prediction of shear modulus, while few present the shear stress–strain behaviour until fracture. Silvestre et al. [30,31] predicted the nonlinear shear response of γ-graphyne complete sheet with the dimensions 10.9 nm × 9:8 nm using uniaxial and biaxial shear tests. Yi et al. [37] performed extensive molecular dynamics simulations on four different graphynes (α-, β-, γ-, and 6,6,12-graphynes) to explore their mechanical properties under shearing and bending. For γ-graphynes, the authors predicted that the fracture shear strength and strain of γ-graphynes differ in the two directions, indicating that the fracture shear strength and strain of graphynes are anisotropic. Specifically, they calculated shear fracture stress of 53.9 GPa and 41.4 GPa in the x and y direction, respectively, for a square γ-graphyne sheet with a side length of approximately 20 nm.

It follows from the foregoing that, although studies on the tensile behaviour of graphene structures are numerous, very little research has been conducted on the nonlinear response of these structures when subjected to shear. To the best of the authors’ knowledge, the present paper may be the first in the available literature to address the behaviour of different types of γ-graphyne structures of different sizes under shearing in a comprehensive way. To achieve this goal, a spring-based finite element method is utilised for the prediction of the whole shear stress–strain mechanical response and the corresponding both linear and nonlinear arising mechanical properties.

## 2. Computational Modelling

This section presents the structural geometry for different γ-graphyne structures in nanoscale, the force field describing the interatomic interactions in the structure, as well as the finite element technique utilised for the prediction of the behaviour of structures under shearing.

### 2.1. Geometry of γ-Graphyne Structures

**Figure 1:**
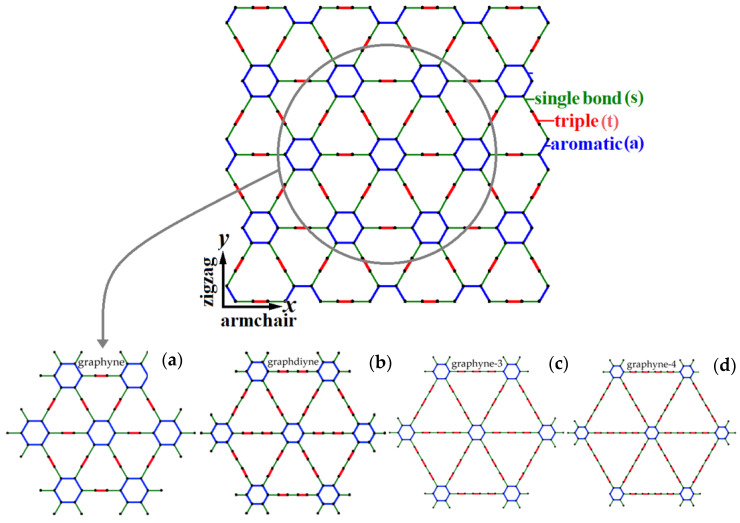
The structural geometry graphyne, graphdiyne, graphyne-3, and graphyne-4.

The γ-graphynes is a group of 2D carbon allotropes composed of sp and sp^2^ hybridised carbon atoms. These graphene-like nanosheets consist of aromatic hexagonal rings and acetylenic groups. Depending on the nanostructural architecture and the combination of the single (C–C), aromatic (C=C), and triple (C≡C) bonds different types of γ-graphyne sheets may be obtained. Here, the types of this graphyne group that were computationally investigated include graphyne-1 (graphyne), graphyne-2 (graphdiyne), graphyne-3, and graphyne-4, presented in Figure 1a–d, respectively, using a global Cartesian coordinate system (*x*,*y*). The aromatic bonds are obviously observed in the hexagonal molecules, while the single and triple bonds are found in acetylenic links. It is apparent that the length of the acetylenic links, controlled by the containing number of single and triple bonds, specifies the naming of the graphyne sheet.

### 2.2. Force-Field and Interatomic Interactions

Along with the molecular mechanics, the total potential energy of the graphyne molecular systems can be presented as the sum of energies due to the interatomic interactions. Given that nonbonded interactions are negligible for the size of the molecular systems investigated here and that only the in-plane properties were to be examined, the total potential energy of a structure of the γ-graphyne group is expressed as

(1) $$U_{\mathrm{tot}}=\sum U_{r}^{b}+\sum U_{\theta }^{b,\beta },$$

where both superscript b and β denote the bond type and take the string values s, a, and t when referring to the simple, aromatic, and triple bond, respectively. The $U_{r}^{b}$ and $U_{\theta }^{b,\beta }$ represent the potential energy terms due to the length change in the bond and bending angle change between the two linked bonds b and β, and for their representation, the following functions were adopted, respectively:

(2) $$U_{r}^{b}=D_{e}^{b}{\left\{[1-e^{-{Β}^{b}(r^{b}-r_{0}^{b})})\right]}^{2}-1\},$$

(3) $$U_{\theta }^{b,\beta }=\frac{1}{2}k_{\theta }^{b,\beta }{\left(\theta^{b,\beta }-\theta_{0}^{b,\beta }\right)}^{2}[1+k_{{}_{\mathrm{sextic}}}^{b,\beta }{\left(\theta^{b,\beta }-\theta_{0}^{b,\beta b}\right)}^{4}].$$

Equations (2) and (3) describe the Morse potential field [38]. The parameters $r^{b}$ and $r_{0}^{b}$ in Equation (1) represent the deformed and undeformed bond length, respectively. Furthermore, $D_{e}^{b}$ and $B^{b}$ are force-field constants that are dependent on the nature of the bond b=s, a, t. The parameters $\theta^{b,\beta }$ and $\theta_{0}^{b,\beta }$ in Equation (3) are the bending angles regarding the deformed and initial geometry between two linked bonds b and β, respectively, while the parameters $k_{\theta }^{b,\beta }$ and $k_{\mathrm{sextic}}^{b,\beta }$ are corresponding constants governing the linear and nonlinear force–deformation behaviour regarding the bond angle bending interactions. It should be noted that, usually, in such graphene-like nanostructures, the nonbonded interatomic interactions and the corresponding produced nonlocality have negligible effects on their overall mechanical behaviour and, thus, may be excluded by the mathematical formulation of the problem.

The force field indicated by the above two equations can be straightforwardly described by differentiating the potential function terms, i.e., Equations (2) and (3), with regard to the bond length variation $\Delta r^{b}$ and bond bending angle change $\Delta \theta^{b,\beta }$, respectively:

(4) $$F_{r}^{b}(\Delta r^{b})=\frac{\partial U_{r}^{b}}{\partial (\Delta r^{b})}=2B^{b}D_{e}^{b}(1-e^{-B^{b}\Delta r^{b}})e^{-B^{b}\Delta r^{b}},$$

(5) $$M_{\theta }^{b,\beta }(\Delta \theta^{b,\beta })=\frac{\partial U_{\theta }^{b,\beta }}{\partial {\left(\Delta \theta^{b,\beta }\right)}^{2}}=k_{\theta }^{b,\beta }\Delta \theta^{b,\beta }[1+4\overset{\approx 0}{\overset{︷}{k_{{}_{\mathrm{sextic}}}^{b,\beta }{\left(\Delta \theta^{b,\beta }\right)}^{3}}}]\approx k_{\theta }^{b,\beta }\Delta \theta^{b,\beta },$$

where $F_{r}^{b}$ is the developed axial force between two bonded atoms because of the change in their inter-distance $\Delta r^{b}$, while $M_{\theta }^{b,\beta }$ is the arisen bending moment due to the change in the angle between two linked bonds $\Delta \theta^{b,\beta }$.

### 2.3. Finite Element Formulation

Equations (4) and (5) indicate that the force field established within the γ-graphyne structure could be simulated by employing a suitable combination of straightforward mechanical analogues. Specifically, each bond length variation or bond angle bending interatomic interaction could be efficiently represented using a translational and a rotational spring, respectively. The longitudinal stiffness of the necessary translational springs may well be defined by differentiating Equation (4) with respect to the bond length variation $\Delta r^{b}$:

(6) $$\frac{\partial F_{r}^{b}}{\partial \Delta r^{b}}=2{\left(B^{b}\right)}^{2}D_{e}^{b}(2-e^{B^{b}\Delta r^{b}})e^{-2B^{b}\Delta r^{b}}=k_{r}^{b}(\Delta r^{b}).$$

Similarly, the necessary rotational stiffness of the rotational spring can be derived via the differentiation of Equation (5) in regard to the bending angle variation $\Delta \theta^{b,\beta }$ as follows:

(7) $$\frac{\partial M_{\theta }^{b,\beta }}{\partial \Delta \theta^{b,\beta }}\approx k_{\theta }^{b,\beta }.$$

**Figure 2:**
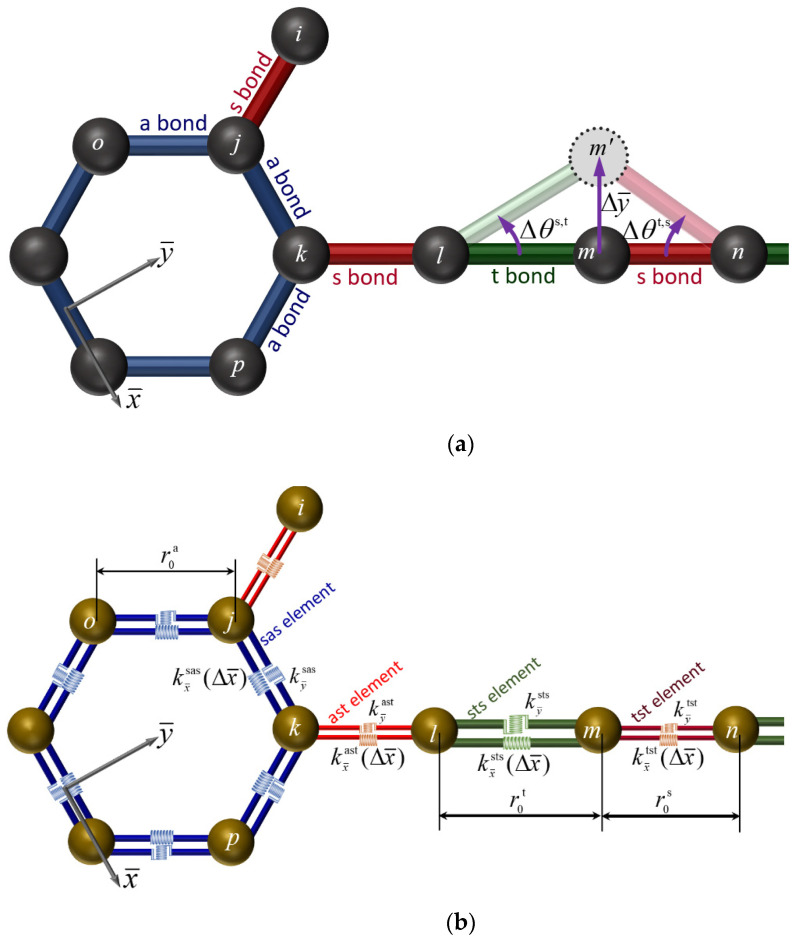
Indicative nanostructure of graphyne: (**a**) configuration of bonds and (**b**) spring-like finite element representation.

In accordance with the presented finite element formulation, the potential energies due to the bond length and bending angle variations are simulated by the use of two-noded, straight, spring-like line-finite elements interconnecting two bonded carbon atoms. The stiffness matrix of the proposed elements in addition to their force–displacement response is derived by a local Cartesian coordinate system ($\bar{x},\bar{y}$), which is presented in Figure 2. As Figure 2 illustrates, their local coordinate system origin is positioned at the centre of the line that connects the two bonded atom positions, while the $\bar{x}$-axis is aligned with this linking line and, thus, passes through its both nodes. The proposed joint finite element is expressed by a 2 × 2 stiffness elemental matrix, which includes two basic coefficients with respect to the $\bar{x}$ and $\bar{y}$ directions.

(8) $$k^{\mathrm{el}}=\left[\begin{array}{cccc} k_{\bar{x}}^{\mathrm{el}} & 0 & -k_{\bar{x}}^{\mathrm{el}} & 0\\ 0 & k_{\bar{y}}^{\mathrm{el}} & 0 & -k_{\bar{y}}^{\mathrm{el}}\\ -k_{\bar{x}}^{\mathrm{el}} & 0 & k_{\bar{x}}^{\mathrm{el}} & 0\\ 0 & -k_{\bar{y}}^{\mathrm{el}} & 0 & k_{\bar{y}}^{\mathrm{el}} \end{array}\right].$$

where the notation el of the element obtains the four different string values sas, ast,  sts, and tst meaning that the present formulation needs the implementation of four types of spring-like elements having different longitudinal and transverse stiffness coefficients, as Figure 2b demonstrates. For instance, the notation  sts relates to a spring-like element, which was employed for the description of a triple bond placed between two single bonds.

According to the local coordinate system of the elements, it is true that $\Delta r^{s}=\Delta r^{a}=\Delta r^{t}=\Delta \bar{x}$. Thus, the nonlinear axial stiffness coefficients of these elements may be defined by using Equation (9) as follows:

(9) $$k_{\bar{x}}^{\mathrm{el}}=\left\{\begin{array}{c} \;2{\left(B^{s}\right)}^{2}D_{e}^{s}(2-e^{B^{s}\Delta \bar{x}})e^{-2B^{s}\Delta \bar{x}},\;\mathrm{el}=\mathrm{ast},\;\mathrm{tst}\\ 2{\left(B^{a}\right)}^{2}D_{e}^{a}(2-e^{B^{a}\Delta \bar{x}})e^{-2B^{a}\Delta \bar{x}},\;\mathrm{el}=\mathrm{sas}\\ 2{\left(B^{t}\right)}^{2}D_{e}^{t}(2-e^{B^{t}\Delta \bar{x}})e^{-2B^{t}\Delta \bar{x}},\;\mathrm{el}=\mathrm{sts} \end{array}\right..$$

According to the simplified modeling technique followed here, provided in detail in [39], the bond angle bending variation can be efficiently treated by solely using an appropriate stiffness coefficient in the $\bar{y}$-direction for each finite element el=sas, ast, sts, tst, given by

(10) $$k_{\bar{y}}^{\mathrm{el}}=\left\{\begin{array}{c} (2k_{{}^{\theta }}^{a,s}+k_{{}^{\theta }}^{s,t}){\left(r_{0}^{s}\right)}^{-2},\;\mathrm{el}=\mathrm{ast}\\ 2k_{{}^{\theta }}^{s,t}{\left(r_{0}^{s}\right)}^{-2},\;\;\;\;\mathrm{el}=\;\mathrm{tst}\\ (2k_{{}^{\theta }}^{a,a}+2k_{{}^{\theta }}^{s,a}){\left(r_{0}^{a}\right)}^{-2},\;\mathrm{el}=\mathrm{sas}\\ 2k_{{}^{\theta }}^{s,t}{\left(r_{0}^{t}\right)}^{-2},\;\;\;\;\;\mathrm{el}=\mathrm{sts} \end{array}\right..$$

To examine the elastoplastic performance of a γ-graphyne sheet, one can write the equilibrium expression for each assumed nonlinear spring-like finite element as

(11) $$k^{\mathrm{el}}u^{\mathrm{el}}=f^{\mathrm{el}}.$$

where $u^{\mathrm{el}}$ and $f^{\mathrm{el}}$ are the elemental displacement and force vector, respectively. If i and j are the two nodes of the finite element, these vectors can be written as

(12) $$u^{\mathrm{el}}={\left[\begin{array}{cccc} u_{\bar{x}i}^{\mathrm{el}} & u_{\bar{y}i}^{\mathrm{el}} & u_{\bar{x}j}^{\mathrm{el}} & u_{\bar{y}j}^{\mathrm{el}} \end{array}\right]}^{T},$$

(13) $$f^{\mathrm{el}}={\left[\begin{array}{cccc} f_{\bar{x}i}^{\mathrm{el}} & f_{\bar{y}i}^{\mathrm{el}} & f_{\bar{x}j}^{\mathrm{el}} & f_{\bar{y}j}^{\mathrm{el}} \end{array}\right]}^{T}.$$

Transforming the elemental stiffness equation for every finite element to the global coordinate system, the final system of nonlinear equations may be assembled, according to the requirements of nodal connectivity, into the following form:

(14) $$K^{\mathrm{el}}(U^{\mathrm{el}})\;U^{\mathrm{el}}=F^{\mathrm{el}},$$

where $K^{\mathrm{el}}{\left(U\right)}^{\mathrm{el}}$, $U^{\mathrm{el}}$, and $F^{\mathrm{el}}$ is the assembled deformation-dependent stiffness matrix, assembled displacement vector, and assembled force vector.

Applying the boundary conditions, i.e., appropriate supports and loads, in order to simulate shearing, a standard incremental–iterative procedure based on the Newton–Raphson algorithm was applied to numerically manage the inherent nonlinearity of the global stiffness matrix and attribute the corresponding performance of the structure. More details about the fundamentals regarding the computational implementation of the proposed finite element-based scheme may be found elsewhere [39,40].

## 3. Results and Discussion

A number of 2D finite element models were established for the graphyne, graphdiyne, graphyne-3, and graphyne-4 case. A total of 20 almost-square models of different sizes and types were developed and analysed under shearing loads along the x (armchair) and y (zigzag) direction, as illustrated in Figure 1d. The size of each 2D nanosheet is characterised by the side length. For small sheet dimensions, the nonlinear mechanical behaviour under shear loadings seems to be highly size-dependent. Table 1 provides the relevant information and details about the geometric and computational characteristics of the structures that were investigated in this study.

**Figure 3:**
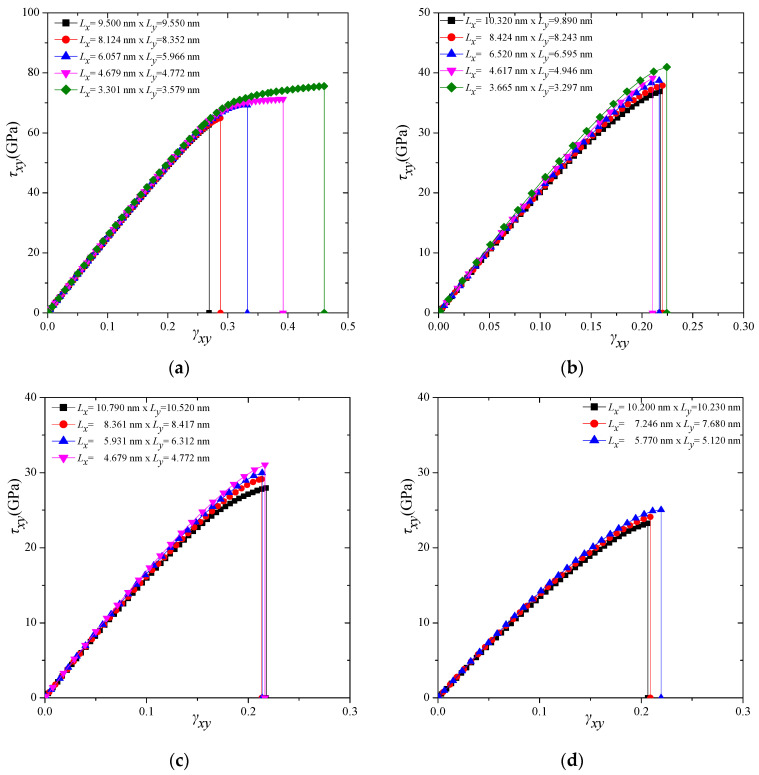
Shear stress–strain response of (**a**) graphyne, (**b**) graphdiyne, (**c**) graphyne-3, and (**d**) graphyne -4 structures in *x–y* direction.

**Figure 4:**
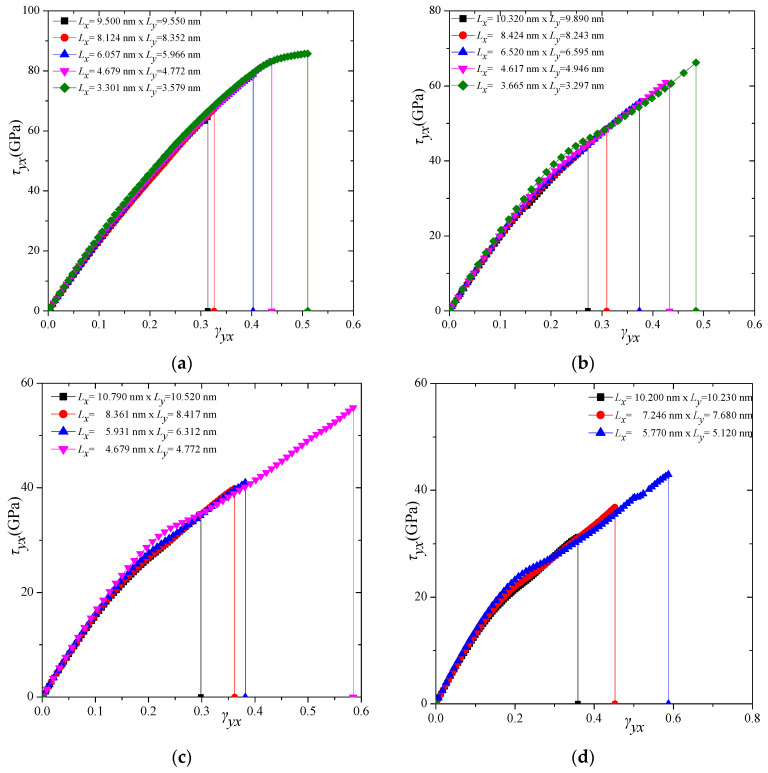
Shear stress–strain response of (**a**) graphyne, (**b**) graphdiyne, (**c**) graphyne-3, and (**d**) graphyne-4 structures in *y–x* direction.

To provide insight regarding the nature of the shear stress–strain curves of the four different γ-graphyne types, Figure 3 depicts the shear stress–strain variations in the almost square-shaped graphyne sheets, only along the *x–y* direction, for graphyne (Figure 3a), graphdiyne (Figure 3b), graphyne-3 (Figure 3c), and graphyne-4 (Figure 3d), respectively. This figure demonstrates that the graphyne is clearly stiffer and of higher strength in shear strain than the other types. Furthermore, there is obviously a dependence of the shear stress–strain response on the size of the structure. In all cases, it seems that the smaller structures present a stronger response than the larger ones. Similarly, Figure 4 depicts the shear stress–strain variations in the almost square-shaped graphyne sheets, only along the *y–x* direction, for graphyne (Figure 4a), graphdiyne (Figure 4b), graphyne-3 (Figure 4c), and graphyne-4 (Figure 4d), respectively. Analogous trends but different behaviours, in general, are observed for the γ-graphyne structures in the *y–x* direction, compared with the *x–y* direction.

**Figure 5:**
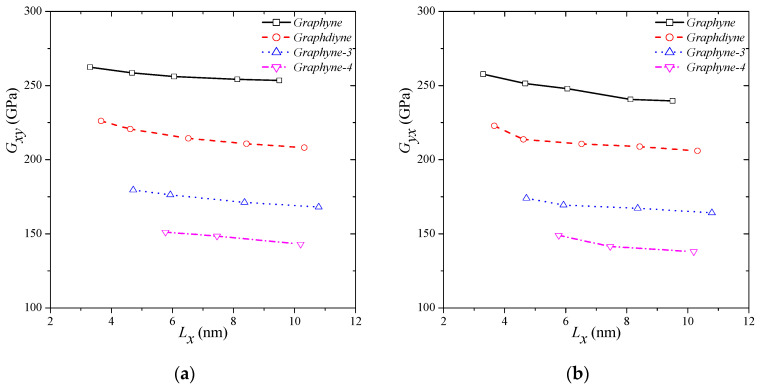
Shear modulus variations in different graphyne structures in (**a**) *x–y* and (**b**) *y–x* direction.

Figure 5 depicts the variations in the shear moduli due to different sizes for all the types of graphyne structures that were investigated in this study. In Figure 5a, the size and type-dependent behaviour of shear modulus in the *x–y* direction is clearly described. The smaller the dimensions are, the higher is the *Gxy*. The highest stiffness is reported for γ-graphyne and is equal to 262.44 GPa for a side length of almost 3.5 nm (3.30 nm in *x*-direction and 3.58 nm in *y*-direction). The lowest stiffness is obtained for graphyne-4 and is equal to 143.10 Gpa for a side length of almost 10 nm (10.20 nm in *x*-direction and 10.23 nm in *y*-direction). Similar observations are noted also for the behaviour of shear modulus in the *y–x* direction. The highest stiffness is reported for γ-graphyne and is equal to 257.68 Gpa for a side length of almost 3.5 nm, and the lowest stiffness is computed for graphyne-4 and is equal to 137.92 Gpa for a side length of almost 10 nm. It is observed that the shear stiffness of graphyne structures is slightly lower in the *y–x* direction than the *x–y* direction.

**Figure 6:**
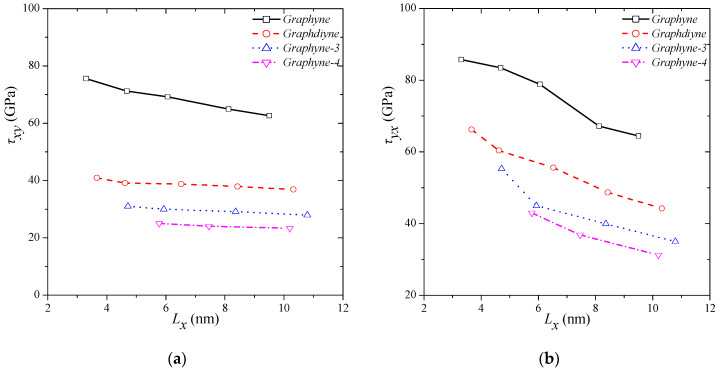
Shear modulus variations in different graphyne structures in (**a**) *x–y* and (**b**) *y–x* direction.

Figure 6 presents the variations in the shear strength for all the types of graphyne structures for different structure dimensions. The smaller the dimensions are, the higher are the *τxy* and *τyx*. In Figure 6a, the strong size- and type-dependent behaviour of shear strength in the *x–y* direction is observed. The maximum strength is reported for γ-graphyne and is equal to 75.60 GPa for a side length of almost 3.5 nm. The smallest strength is calculated for graphyne-4 and is equal to 23.34 GPa for a side length of almost 10 nm. Comparable results are also observed for the behaviour of shear strength in the *y–x* direction. The highest strength is reported for γ-graphyne and is equal to 85.74 GPa for a side length of almost 3.5 nm, and the lowest strength is computed for graphyne-4 and is equal to 31.23 GPa for a side length almost 10 nm. It is noted that the shear strength of graphyne structures is clearly higher in the *y–x* direction than the *x–y* direction. The values of shear strength seem to be consistent with the shear strength values of graphene, i.e., 100–120 GPa [40], respectfully following a certain tendency. As the number of single–triple–single configurations increases, and therefore, the acetylenic linkage becomes longer, the shear moduli and strength decreases.

**Figure 7:**
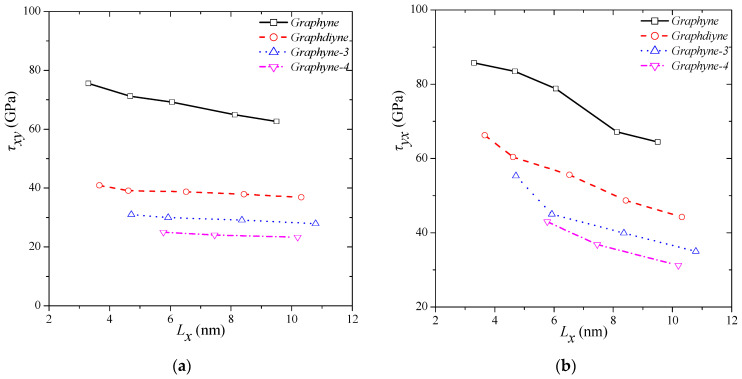
Shear strength variations in different graphyne structures in (**a**) *x–y* and (**b**) *y–x* direction.

Figure 7 presents the behaviour of the shear fracture strain versus the structure size for all the types of graphyne structures. The smaller the dimensions, the higher the *γxy* and *γyx*. The size- and type-dependent behaviours of shear fracture strain in the *x–y* direction were examined, the results of which are presented in Figure 7a. The greatest fracture strain is achieved for γ-graphyne and is equal to 0.46 for a side length of almost 3.5 nm. The smallest fracture strain is computed for graphdiyne and is equal to 0.208 for a side length of almost 10 nm. For higher sizes, all structure types seem to converge at a specific shear fracture strain range 0.20–0.25. Comparable results are computed for variations in shear fracture strain in the *y–x* direction too. The highest fracture strain is reported for γ-graphyne and is equal to 0.588 for a side length almost 5.5 nm, and the lowest fracture strain is calculated for graphdiyne and is equal to 0.272 for a side length almost 10 nm. It is noted that the fracture strength of graphyne structures is clearly higher in the *y–x* direction than the *x–y* direction. For lower sizes, the fracture strain range is about 0.55 ± 0.05, and for higher sizes, all structure types seem to converge at a specific shear strain range of 0.31 ± 0.05 (side length ~ 10 nm). These values of shear fracture strain are also predicted for square graphene of 10 nm side length [40].

To validate the proposed computational approach concerning the performance of γ-graphynes under shearing forces, Table 2 demonstrates various comparisons between the results of the present numerical technique and estimations gathered from different approaches found in the literature. A reasonable agreement with the results of the other published studies may be observed. Differences observed may be occurred due to the size dependency of the structural mechanical response. Concerning the nonlinear results for graphynes imposed to shear loading, the published data are limited to a few studies concerning only graphyne and for specific sizes, without any analysis about the size effect. More results can be found about the elastic properties of graphyne structures, and the comparisons about the shear stiffness performed to demonstrate the accuracy of the proposed method.

## 4. Conclusions

A nonlinear finite element method was presented for the modelling of shear stress–strain response of γ-graphyne structures, i.e., graphyne, graphdiyne, graphyne-3, and graphyne-4, up to their fracture, for a range of geometric characteristics. To model the nanostructures, nonlinear, spring-like finite elements were employed to model the interatomic interactions between carbon atoms. From the stress–strain variations, essential material properties such as shear modulus, shear fracture stress, and shear fracture strain were revealed. As a general conclusion, graphynes have higher shear stiffness and shear strength than the other types of the family, and all the structures present size-dependent behaviour. The properties seem to become gradually less for higher sizes. The proposed approach, through comparisons where it was possible, is proved to be an effective and sufficiently accurate tool for the characterisation of graphyne structures and potentially other similar nanostructures.

## Figures and Tables

**Table 1:** Information regarding the utilised FEM models.

Type	Side Length ~ (nm)	Lx (nm)	Ly (nm)	Total Number of Nodes	Total Number of Elements
Graphyne	3.5	3.30	3.58	404	930
	4.5	4.68	4.77	748	1738
	6.0	6.06	5.97	1196	2794
	8.0	8.12	8.35	2218	5210
	10	9.50	9.55	2952	6948
Graphdiyne	3.5	3.67	3.30	304	686
	4.5	4.62	4.95	558	1270
	6.0	6.52	6.60	1034	2368
	8.0	8.42	8.24	1654	3802
	10	10.32	9.89	2418	5572
Graphyne-3	4.5	4.71	4.21	404	884
	6.0	5.93	6.31	742	1636
	8.0	8.36	8.42	1376	3050
	10	10.79	10.52	2202	4895
	5.5	5.77	5.12	504	1084
Graphyne-4	7.5	7.46	7.68	926	2004
	10	10.20	10.23	1718	3734

**Table 2:** Some comparisons between the present numerical outcome and other corresponding data.

Type	Method	Side Length~(nm)	*Gxy* (N/m)	*Gyx* (N/m)	*τxy* (N/m)	*γxy*	*τyx* (N/m)	*γyx*
Graphyne	Present	10	81.10	76	24.19	0.268	20.62	0.313
	MD [37]	20	85.60	67.2	13.28	0.143	17.24	0.153
	DFT [41]	15	77.04			0.18		
	FE [30]	10	40.70		4.6	0.18	5.5	0.16
	FE [36]	10	70.50			0.1145		0.1147
	DFT [13]	12	57.64					
	FE [31]	10	56.53		10.4	0.25		
	MD [42]	12			15.7 (Gpa)	0.07	23.1 (Gpa)	0.246
Graphdiyne	Present	10	66.61	65.69	11.81	0.208	14.16	0.272
	DFT [13]	12	43.18					
